# Efficacy of *Clonostachys rosea* and *Duddingtonia flagrans* in Reducing the *Haemonchus contortus* Infective Larvae

**DOI:** 10.1155/2015/474879

**Published:** 2015-10-04

**Authors:** Manoel Eduardo da Silva, Fabio Ribeiro Braga, Pedro Mendoza de Gives, Miguel Angel Mercado Uriostegui, Manuela Reyes, Filippe Elias de Freitas Soares, Lorendane Millena de Carvalho, Francielle Bosi Rodrigues, Jackson Victor de Araújo

**Affiliations:** ^1^Departamento de Veterinária, Universidade Federal de Viçosa, 36570000 Viçosa, MG, Brazil; ^2^URECO/EPAMIG, 35650000 Pitangui, MG, Brazil; ^3^Universidade Vila Velha, Vila Velha, ES, Brazil; ^4^Área de Helmintologia, CENID-Parasitologia, INIFAP, Jiutepec, MOR, Mexico; ^5^Departamento de Microbiología Ambiental y Biotecnología, UAC, CAM, Mexico; ^6^Programa de Pós Graduação em Ciências Farmacêuticas (PPGF-UVV), Universidade Vila Velha, 29102920 Vila Velha, ES, Brazil

## Abstract

The biocontrol is proven effective in reducing in vitro and in situ free-living stages of major gastrointestinal helminths, allowing progress in reducing losses by parasitism, maximizing production, and productivity. This study aimed at evaluating the predatory activity of fungal isolates of* Duddingtonia flagrans *and* Clonostachys rosea *species and its association on infective larvae (L_3_) of* H. contortus *in microplots formed by grasses and maintained in a protected environment. All groups were added with 10 mL of an aqueous suspension with 618* H. contortus* L_3_ approximately. Group 1 was used as control and only received the infective larvae. Groups 2 and 3 received* D. flagrans* chlamydospores and* C. rosea *conidia at doses of 5 × 10^6^. Group 4 received the combination of 5 × 10^6^ *D. flagrans* chlamydospores + 5 × 10^6^ *C. rosea* conidia.* D. flagrans *and* C. rosea* showed nematicidal effectiveness reducing by 91.5 and 88.9%, respectively, the population of* H. contortus *L_3_. However, when used in combination efficiency decreased to 74.5% predation of* H. contortus *L_3_. These results demonstrate the need for further studies to determine the existence of additive effects, synergistic or antagonistic, between these species.

## 1. Introduction

Endoparasitic nematodiasis are considered as one of the main factors seriously affecting the livestock industry, since they cause an important reduction not only in the animal weight but also their main products; that is, meat and milk are also diminished [1]. Other problems derived from nematode parasitic infections are the continuous expenses in chemical treatments and eventually the death of young animals [2]. The use of chemical drugs is so far the unique method of anthelmintic control that is established around the world [3]. Unfortunately the use of drugs and other measures of control is in most of the cases not based on the adequate recommendations, that is, the general animal state, body weight, diagnosis of parasitic burden, and other important factors that should be considered to establish a successful control as quantity and quality of food, selection of gastrointestinal parasitic genetically resistant animals, and the presence of parasites resistance to chemical drugs [4].

A number of alternatives of control different than the use of chemical drugs are being proposed, although the most promising measure of control is the use of natural nematode antagonists like nematophagous fungi [5, 6]. Allied to chemical control, one of the most promising and sustainable methods of control is biological control, which has shown a good effectiveness either in laboratory trials or in pasture experiments which is an important activity reducing the free-living stages of mayor gastrointestinal helminths. This method can be considered as a useful tool of control which reduces the economic losses caused by parasites and can promote a higher animal productivity [7, 8].

The species* Clonostachys rosea* belonging to the order Hypocreales (family: Bionectriacea) has being recorded as a parasite of other microfungi and also as a nematode parasite, apart from being a saprophyte fungus [9]. This species colonizes plants and it has attracted interest by workers as a potential biological control agent, since it produces volatile organic compounds toxic for some living organisms [10, 11].

*Duddingtonia flagrans* is an “old friend” of scientists who envision biological control as an alternative to the treatment of herd animals by chemical means and also in the future of prevention of high levels of contamination by nematodes in public areas and squares used by humans [3, 12]. Again it must be remembered here that the ability to pass through the gastrointestinal tract of domestic animals makes this species of fungus an eternal ally in combating nematodes present in the environment [13].

This fungal species is said to be the “most promising.” Its main focus of activity culminates in the predation of infective helminth forms (in this case, nematode larvae), by means of simple adhesive hyphae.

The fungus* D. flagrans* belonging to the order Moniliales (family: Moniliaceae) is one of the most promising microorganisms for biological control of ruminant parasitic nematodes [14]. This species is harmless to plants, animals, and human being [15, 16] and it is possible to isolate it mainly from soil samples and animal faeces [17]. This microorganism possesses dual food habits, being saprobius in absence of nematodes and becoming either predatory or parasite of nematodes in the presence of them [18].

Taking into account the representation of the world livestock and its socioeconomics, it is important to evaluate, improve, and provide the use of technologies that can contribute to the development of this segment of agrobusiness. The aim of this study was to evaluate the predatory activity of fungal isolates of the species* C. rosea* and* D. flagrans* and their association on* H. contortus* infective larvae (L_3_) in microplots with graminaceous forage plant.

## 2. Material and Methods

### 2.1. Fungi

The* C. rosea* Yucatan strain, belonging to the CICY collection, was used. This strain was maintained in 2% water-agar (2% WA) at the Department of Helminthology (CENID-PAVET-INIFAP-MEXICO). The* D. flagrans* fungal strain (FTHO-8) belonging to the Fungal Collection of CENID-Parasitología Veterinaria, INIFAP-Mexico, was used. This strain was originally obtained from a sheep faecal sample from a farm at Fierro del Toro Village, Huitzilac Municipality, Morelos State, Mexico [19].

### 2.2. Nematodes

A* H. contortus* isolate was originally obtained from a naturally infected sheep from “Las Margaritas” Experimental Sheep Farm (INIFAP) in Hueytamalco Municipality in the state of Puebla, Mexico. A* H. contortus *egg donor lamb was orally infected with an aqueous suspension containing 350 larvae/kg of body weight. Twenty days after infection the presence of* H. contortus* eggs in faecal samples was detected through the McMaster technique. Faecal cultures were prepared by mixing faeces with polystyrene particles in plastic bowls. Water was added to the faecal cultures and the mixture was homogenized to keep the cultures hydrated and oxygenated to promote better larval hatching [20]. Faecal cultures were covered with paper foil and incubated for 7 days at room temperature (25–30°C). Infective larvae were extracted from faeces using the Baermann funnel technique [21]. The larvae were washed several times in density gradients of 50% sucrose solution and rinsed and suspended in sterile water.

### 2.3. Assay

The pots were conformed as follows: thirty-six plastic (gardening) pots with a capacity of 100 grams (75 × 55 mm) were used. Seventy grams of soil acquired from commercial nurseries was autoclaved at 1.2 atm and 115°C for 15 minutes and added to the plastic pots. Every pot with soil was added with 0.06 grams of a commercial mixture of different genera/species grass seeds; about 70 graminaceous seeds were added to the soil surface in order to obtain a homogeneous grass growing. Six mL of top water was added to every pot every day during 18 days of the experiment using a Pasteur pipette to maintain the pots humidity.

After germination the pots were randomly divided into four (4) experimental groups with 8 replicates each. All groups were added with 10 mL average of an acqueos suspension with 618* H. contortus* L_3_ approximately. Group 1 was used as control and only received the infective larvae. Groups 2 and 3 received* D. flagrans* chlamydospores and* C. rosea* conidia at doses of 5 × 10^6^, respectively. Group 4 received the combination of 5 × 10^6^ *D. flagrans* chlamydospores + 5 × 10^6^ *C. rosea* conidia.

The pots were maintained under a shading rate hedging protected screen and after 12 days of interaction between nematophagous fungi and parasitic nematodes, the whole nematode population from total soil and grass of every single pot was recovered using the Baermann Funnel method [21].

### 2.4. Location

The experiment was conducted in CENID-PAVET-INIFAP-MEXICO, located at 18°53′04′′ N latitude and 98°51′34′′ W and 1615.16 meters above sea level, in the municipality of Jiutepec, Morelos, Mexico.

### 2.5. Weather Data

Meteorological data including maximum and minimum temperatures, relative humidity, and rainfall during the experiment were obtained from the closest experimental weather station (National Institute for Water Technology), Jiutepec, Morelos, Mexico.

### 2.6. Statistical Analysis

Data were analysed using an ANOVA test and the Tukey test was used as a complementary technique for discriminating the mean different from the others. Data were analysed using the software BioEstat 5.3.

## 3. Results and Discussion

The results are shown in Table 1, which shows the mean, standard deviation, and the* H. contortus* larvae reduction percentage after being recovered from pots after 12 days of interaction between nematophagous fungi and nematode larvae. The average temperature value during the experimental period was 16.1°C, ranging from 15 to 33°C; the environmental relative humidity was 71.03%, ranging from 15.2 to 108% and 7 mm of rain. These environmental conditions are considered as ideal for the development of ruminant parasitic larvae [22] and nematophagous fungi [23].

The results obtained using* D. flagrans* in reducing the nematode larvae population into animal faeces have generated the development of studies focused on the production of chlamydospores which are resistant stages of some microfungi for the control of ruminant parasitic nematodes [13]. The results of the present work are promising and an important reduction in the larval population recovered from treated pots has shown evidence that fungi produce satisfactory results with the use of 5 × 10^6^ conidia and chlamydospores per pot; and although this is only a model of study, it gives a clear image about the potential use of this biological system of control of infective larvae on the contaminated grass.

The recovery larvae mean values have shown statistical difference among the treated groups when compared with control group (*p* < 0.001). However, no differences were found among the treated groups (*p* > 0.005) (Table 1). Such results corroborate those published of De Almeida et al. [24] that demonstrated high effectiveness of the specie* D. flagrans* against different genera of parasites affecting ruminants such as* Haemonchus, Teladorsagia,* and* Trichostrongylus*.

The nematophagous* C. rosea* fungus was able to reduce by 88.9% the number* H. contortus* L_3_.* D. flagrans* was able to reduce by 91.5% the number* H. contortus *L_3_. The association of the micro fungi* D. flagrans* and* C. rosea *was able to reduce 74.5% of* H. contortus* (L_3_) infective larvae.

Higher in vitro reductions (94.21–99.61%) in the* H. contortus* L_3_ population by different* D. flagrans* isolates were recently found by Priego-Cortes [25], although it is important to remark that this research was performed under in vitro conditions on water agar plates. In the same work the in vitro activity of* D. flagrans *isolates, FTHO-8, M3, and DFIPC, showed 99.2, 98.23, and 98.8% reductions against the root-knot nematode* Meloidogyne* sp. Such results are encouraging in searching for a fungal candidate as a potential agent of agricultural pest control.

Assis et al. [5] evaluated the predatory activity of* D. flagrans* and* M. thaumasium* in the control of nematodiasis in grazing cattle by administering fungal pellets obtaining a reduction of 47.8 and 56.67% of the number of larvae, respectively, and these results were lower than those found in our study, although conducted in animals grazing.

In the present study, the nematophagous fungus* C. rosea* reduced by 76.9% the number of* H. contortus* L_3_ under environmental conditions. Similar results were recently obtained using two isolates,* C. rosea* (Yucatán strain) and* Clonostachys* sp.

One Campeche strain* C. rosea* reduced 84.2 and 59.5%* H. contortus* L_3_, respectively. These fungi also showed 94.3 and 95.9% reduction on the phytonematode* Meloidogyne* sp. (J2), respectively [26].

Baloyi et al. [27] evaluated the in vitro predatory activity of* C. rosea* (in faeces and in water) against trichostrongylids larvae and they reported 69.9 and 89.3%, reductions, respectively, of the number of trichostrongylids larvae in bioassays made in sheep feces and water. On the other hand, Rodriguez-Martínez [28] evaluated in vitro predatory activity of* C. rosea* against* Rhabditis* sp.,* Caenorhabditis elegans*,* Panagrellus redivivus, Butlerius* sp., and* H. contortus* and they recorded 71.9, 94.7, 92.7, 100, and 87.7%, larvae reductions, respectively. Their results have shown evidence about the potential of these species in the control of parasites of veterinary importance. These results are similar to those described in the present work performed in soil pots.

Dong et al. [10] showed that the metabolites obtained of* C. rosea* were lethal 24 hours after treatment to 50% of* C. elegans, P. redivivus,* and* B. xylophilus *larvae; in our work we obtained better results of predation for free-living nematodes same test being done in vivo. Other researches in phytopathology highlight the relevance and efficiency of the species* C. rosea* as entomopathogenic against* Cicadellidae hemiptera*, causing, respectively, 82.5 and 45% mortality in* Oncometopia tucumana* and* Tapajosa rubromaginata *at 14 days after incubation [29]. Vega et al. [30] showed that* C. rosea* and* B. bassiana* associated were able to reduce 82.5% of the insects* Hypothenemus hampei* in coffee beans and Carreño-Perez et al. [31] showed that* C. rosea* reduction 79% of diseases caused by plant pathogenic fungus* Phytophthora cactorum* isolated on apple fruits.

According to Ayers et al. [32] a group of compounds extracted from* C. candelabrum* showed 90% of efficacy against* H. contortus*. These compounds were synthesized by Rama Rao [11], the macrolide clonostachydiol derived from* C. cilindrospora* that showed 80–90% of reduction of* H. contortus* in artificially infected sheep when subcutaneously administered at a dose of 2.5 mg/kg, corroborating with 21 our results, although we have worked with environmental control.

The association of the micro fungi* D. flagrans* and* C. rosea* was able to reduce 74.5% of* H. contortus* (L_3_) infective larvae. Furthermore Baloyi et al. [27] demonstrated that the combination of* B. thuringiensis* and* C. rosea* showed an additive effect in vitro causing a mortality of 76.7% of parasites nematode of sheep possibly due to the action of* C. rosea* (enzyme production) and* B. thuringiensis* (endotoxin production).

Some studies have noted the combination of nematophagous fungi under natural conditions to combat parasitic forms (eggs and/or larvae) of gastrointestinal nematode parasites. This association of fungi has been tested and the results are relevant. Tavela et al. (2013) reported that the nematophagous fungi have been used as an alternative for controlling gastrointestinal nematodes from domestic animals under natural and laboratory conditions. At that time, those authors demonstrated that the associations of* D. flagrans* and* M. thaumasium* were viable after passing through the gastrointestinal tract in horses and could be used under natural conditions. However, these authors have stated that it is unclear “whether the association of some of these species could bring some kind of advantage,” from the biological point of view, since the antagonism or synergism between species must be better understood.

In the present study, the association of* Clonostachys* and* D. flagrans* was effective and can be used in new experimental designs under laboratory conditions and in the field.* C. rosea* and* D. flagrans* have shown nematicidal effectiveness when were used alone or in combination, effectively reducing the population of infective larvae (L_3_) of* H. contortus* in controlled environmental conditions.

However, the authors believe that there is a need for further studies to determine the existence of additive, synergistic, or antagonistic effects between these species. It is read here that Braga et al. (2014) discuss the predatory activity of an isolate from the nematophagous fungus* Arthrobotrys robusta*, as an example of predatory activity on L_3_ of* H. contortus*. At that time, the authors demonstrated a percentage reduction of 73.8% by the end of seven days. Thus, in this study, it is suggested that the tested association (*Clonostachys* +* Duddingtonia*) could be further explored in future studies of biological control, since the percentage obtained was 74.5%. This is the first report of* C. rosea* and* D. flagrans* association and is the first report to control* H. contortus* in environmental conditions.

## Figures and Tables

**Table 1:** Number and percentage of reduction of *Haemonchus contortus* L_3_ recovered by the method of Baermann 12 days after interaction with fungal isolates *Clonostachys rosea* and *Duddingtonia flagrans*, and association of these microfungi.

Treatment	Control	*C. rosea *	*D. flagrans *	*D. flagrans* + *C. rosea *
*H. contortus *	118 (±114)^a^	13 (±19)^b^	10 (±15)^b^	30 (±19)^b^
% reduction L_3_	0	88.9%	91.5%	74.5%

^*∗*^Different small letters in rows indicate the existence of a statistical difference (*p* < 0.01).
